# Clinical outcomes of adverse drug reaction-related hospital admissions in older adults with diabetes

**DOI:** 10.3389/fphar.2025.1729848

**Published:** 2026-01-12

**Authors:** Azizah Vonna, Mohammed S. Salahudeen, Gregory M. Peterson

**Affiliations:** 1 School of Pharmacy and Pharmacology, UTAS Health, University of Tasmania, Hobart, TAS, Australia; 2 Department of Pharmacy, Faculty of Mathematics and Natural Sciences, Universitas Syiah Kuala, Banda Aceh, Aceh, Indonesia

**Keywords:** adverse drug reactions, diabetes mellitus, older adult, in-hospital mortality, length of stay, rehospitalisation, clinical outcomes

## Abstract

**Aim:**

This is a retrospective cohort study aimed to investigate the association of adverse drug related (ADR)-related hospital admissions with adverse clinical outcomes, including in-hospital mortality, length of stay (LOS), and hospital readmission among older adults with diabetes.

**Methods:**

All individuals aged 65 years and older with diabetes who were admitted to the three major public hospitals in Tasmania, Australia, between July 2017 and December 2023 were identified using International Classification of Diseases codes. Patients with at least one ADR-related hospital admission were propensity score matched with and those without ADR-related admissions based on age, sex, year of index admission, diagnosis-related groups, socioeconomic status, and comorbidities. Adjusted logistic regression was used to assess in-hospital mortality. Length of stay was analysed using a generalised linear model with a Gamma distribution, and readmission risk at 30, 60, and 90 days was assessed using Cox proportional hazards models.

**Results:**

After matching, a total of 5,038 older patients with diabetes were included in the in-hospital mortality analysis, and 4,674 were included in the LOS and readmission analyses. Patients with ADR-related hospitalisations had a significantly higher risk of in-hospital mortality (adjusted odds ratio = 1.31; 95% CI: 1.03–1.66; p < 0.05), longer LOS (adjusted ratio = 1.24 (1.13–1.37); p < 0.001, and a greater risk of readmission (the highest adjusted hazard ratio was at 60 days = 1.29 (1.14–1.45); p < 0.001) compared to those without ADR-related hospital admissions.

**Conclusion:**

ADR-related hospital admissions were associated with poorer clinical outcomes in older adults with diabetes, including greater mortality, prolonged hospital stays, and increased risk of readmission. These findings underscore the importance of early ADR detection, structured medication review, ongoing monitoring, and patient-centered education to improve medication safety and optimise outcomes in this high-risk group.

## 1 Introduction

Adverse drug reactions (ADRs) are a persistent challenge to patient safety worldwide and represent a major contributor to morbidity and mortality (Le Louët and Pitts, 2023). A systematic review involving approximately 1.57 million patients reported that ADRs occurred in 8.32% (95% CI, 7.82, 8.83) of primary care patients during the observation periods of the included studies, with over 20% of these ADRs considered preventable (Insani et al., 2021). A previous systematic review in older adults reported that 3.3%–23.1% of all hospital admissions were attributable to ADRs (Cosgrave et al., 2025). These events represent a significant subset of medication-related problems with substantial implications for healthcare systems.

In Australia, an estimated 250,000 medication-related events result in hospital admissions annually, imposing an economic burden of around A$1.4 billion on the healthcare system (Lim et al., 2022). Reported rates of hospital admissions attributable to ADRs vary widely, ranging from 0.16% to 23.1% of total admissions (Kongkaew et al., 2008; Oscanoa et al., 2017; Howard et al., 2007). This variation reflects differences in study design, population characteristics, and healthcare settings. Risk factors commonly associated with ADRs include advanced age, with age-related physiological changes (e.g., impaired renal function), and polypharmacy (Cahir et al., 2023; Cheong et al., 2020; Pont et al., 2014; Yadesa et al., 2021).

Older adults with diabetes are particularly susceptible to ADRs due to multiple compounding factors. These include the chronic medical complications of diabetes and resultant co-morbidities (Chinmayee et al., 2024; Lu et al., 2023), often necessitating complex medication regimens (Remelli et al., 2022; Sinclair and Abdelhafiz, 2022; Umegaki, 2024). The elevated risk is also partly attributed to diabetes-related physiological changes, such as altered gastric emptying time that can affect drug absorption, and nephropathy, that can impair drug elimination (Dostalek et al., 2012).

Additionally, Australian studies have reported that patients with diabetes who are admitted to hospital for any reason are more likely to have poor clinical outcomes and increased hospital costs, compared to patients without diabetes (Gao et al., 2021; Karahalios et al., 2018). Despite this, evidence regarding the clinical impact of ADR-related hospital admissions in older adults with diabetes remains scarce. Previous studies have examined ADRs in general populations or in other specific conditions, such as dementia (Patel and Patel, 2018; Zaidi et al., 2021; Walter et al., 2017), but none, to our knowledge, have specifically evaluated mortality, length of stay (LOS), and readmission outcomes in older adults with diabetes who experience ADR-related admissions. Therefore, this study aimed to investigate the association between ADR-related hospital admissions and key clinical outcomes, such as in-hospital mortality, LOS, and hospital readmission, among older adults with diabetes.

## 2 Methods

### 2.1 Data source

We conducted a retrospective, population-based cohort study using an administrative dataset routinely collected and reported for patients admitted to hospitals in Australia (Australian Institute of Health and Welfare, 2024). The data set, Admitted Patient Care Australian National Minimum Data Set (APC-NMDS), captures summarised information on all hospital admissions. This incorporates patients’ demographics, admission characteristics, and both principal and additional diagnoses in accordance with the International Statistical Classification of Diseases and Related Health Problems, 10th edition, Australian Modification (ICD-10-AM). Details about the dataset and the data preparation process have been previously described (Vonna et al., 2025).

### 2.2 Study population

Firstly, older patients (aged 65 years and older) who were admitted to the three major public hospitals in Tasmania, Australia from July 2017 to December 2023 were identified. Eligible patients were Tasmanian residents with at least one overnight hospital stay. The presence of diabetes was identified using ICD-10-AM codes E10–14, flagged as either a principal or secondary diagnosis, and encompassing both type 1 and type 2 diabetes.

Patients were divided into two groups based on whether they had any ADR-related hospital admissions, considering both primary and secondary diagnoses. Similar to other studies (Walter et al., 2017; Du et al., 2017), we identified patients with ADR-related hospital admissions using the ICD-10-AM external cause codes between Y40-Y59 or ADR-related diagnosis codes (A1 and A2). The index admission was defined as the first ADR-related hospital admission during the study period for patients who experienced at least one ADR-related admission, or the earliest hospital admission during the study period for those without any ADR-related admission. Demographic and clinical characteristics, as well as in-hospital mortality and LOS, were obtained from this index admission. Readmission analyses were conducted based on outcomes following discharge from the index admission.

### 2.3 Covariates

Covariates included age, sex, year of index admission, socioeconomic status (SES), hospital, Australian Refined Diagnosis Related Groups (AR-DRG), Charlson Comorbidity Index (CCI) score and mode of admission (acute and non-acute). SES was assessed using one of the components of Socioeconomic Indexes for Areas (SEIFA), which is the Index of Relative Socioeconomic Advantage and Disadvantage (IRSAD). Based on the 2016 ABS census data, IRSAD ranks residential postcodes from 1 (most disadvantaged) to 10 (most advantaged) (Australian Bureau of Statistics, 2025). Patients in the lower half of the IRSAD scale (deciles 1–3) were classified as having disadvantaged SES, while those in the upper half of the IRSAD scale (deciles 4–10) were assigned to advantaged SES. The CCI score was calculated using the method described by Quan et al. (Vonna et al., 2025), modified to exclude age and diabetes since all patients were older adults diagnosed with diabetes. The AR-DRG system classifies inpatient admissions based on principal and secondary diagnoses, procedures, patient demographics, and admission/discharge characteristics (IHPA Independent Hospital Pricing Authority, 2025). For analysis, AR DRGs were grouped into twelve categories representing organ system disorders.

### 2.4 Adverse clinical outcomes

We evaluated three primary outcomes associated with ADR-related hospital admissions: (1) in-hospital mortality and (2) LOS (in days) during the index admission, and (3) all-cause rehospitalisation within 30, 60, and 90 days after discharge.

### 2.5 Propensity score matching

Propensity score matching (PSM) was applied to balance baseline covariates between patients with and without an ADR-related hospital admission during the study period, to investigate the association between such admissions and adverse clinical outcomes (Australian Institute of Health and Welfare, 2024). Prior to finalising the propensity score model for matching, multicollinearity among covariates was assessed using correlation coefficients and variance inflation factors (VIF). Covariates with correlation coefficients above 0.8 (or below −0.8) and VIF >2 were excluded from the model. Nearest-neighbour matching with a 1:1 ratio, and a caliper width of 0.1 on the logit of propensity score without replacement was used for matching (Australian Institute of Health and Welfare, 2024).

**FIGURE 1:**
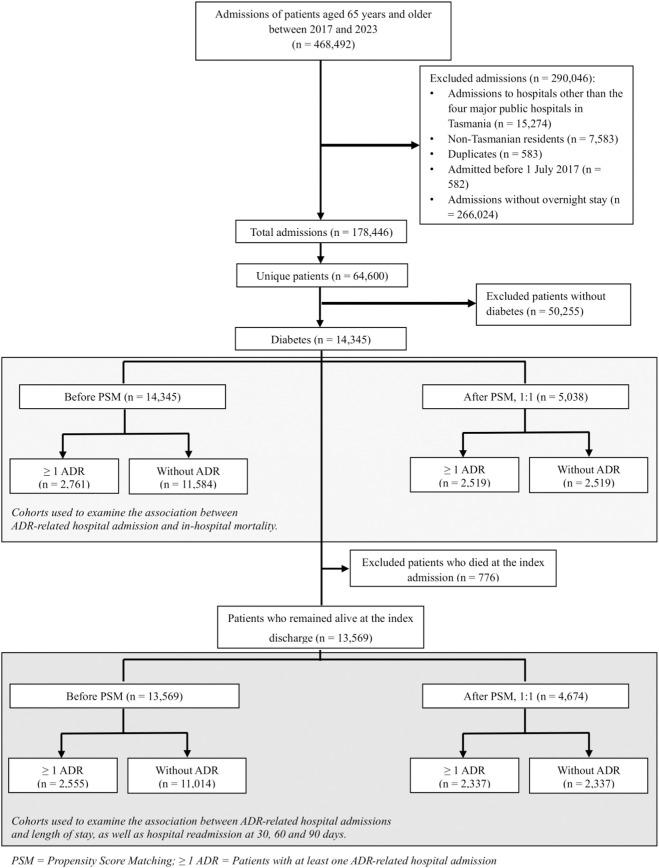
Patient selection flow chart.

PSM was conducted twice: first on the total cohort and then on a subset. The total cohort included all patients, regardless of their survival status, and was used for in-hospital mortality analysis. The second PSM was applied to a subset of patients who survived the index hospital admission, this subset was used for LOS and rehospitalisation analyses (Figure 1).

### 2.6 Statistical analysis

Baseline characteristics were summarised using descriptive statistics and were compared between unmatched (pre-PSM) and matched (post-PSM) groups for both the total cohort and the subset. Matching was considered successful for each characteristic if the standardised mean difference (SMD) between the cohorts was <0.1.

We used logistic regression to calculate odds ratios (ORs) for the association between ADR-related hospital admission and in-hospital mortality using the total cohort data. Only patients who survived the index hospital admission were included in the analyses of LOS and hospital readmission. LOS during the index admission was analysed using a generalised linear model (GLM) with a Gamma distribution and a log link, which is appropriate for modelling positively skewed, continuous outcomes, such as LOS (Du et al., 2017). Cox proportional hazards models were used to estimate hazard ratios (HRs) for all-cause rehospitalisation at 30, 60, and 90 days after index discharge. As a visual exploratory analysis, we also plotted stratified Kaplan–Meier survival curves to estimate survival (no rehospitalisation) at 30, 60, and 90 days. Based on clinical relevance, all models were adjusted for age (as a continuous variable), gender, SES, year of admission, hospital, CCI score (as a continuous variable), admission source, AR-DRG, and the total number of hospital admissions during the study period (as a continuous variable). The study followed the Strengthening the Reporting of Observational studies in Epidemiology (STROBE) checklist for cohort studies (see Supplementary Table S1) (Vandenbroucke et al., 2007).

All statistical analyses were performed using R version 3.6.2 (R Foundation for Statistical Computing, Vienna, Austria), employing the “Matching” package for PSM, the glm () with a logit link for OR estimation, the lm () for ratio analysis, coxph () for HR analysis, and survfit () for Kaplan–Meier survival analysis (Australian Institute of Health and Welfare, 2024).

### 2.7 Ethics

Ethics approval was obtained from the Tasmanian Human Research Ethics Committee (Reference: H0026248).

## 3 Results

Patient inclusion is shown in Figure 1. Among 14,345 older adults with diabetes admitted during the study period, 2,761 (19.2%) experienced at least one ADR-related hospital admission. Most patients were diagnosed with type 2 diabetes (96.7%). The median age of the overall cohort was 76 years (IQR: 70–82), 56% were male, and 59% were classified as socioeconomically disadvantaged. The median number of hospital admissions per patient was 2 (IQR:1–3), and the median CCI score was 1 (IQR: 0–3). Detailed descriptions of patient demographics and ADR characteristics are reported elsewhere (Vonna et al., 2025). Among those with ADR-related admissions, the three most common AR-DRGs were digestive (17%), respiratory (14%), and 151 circulatory (13%) disorders (see Table 1 for details on the characteristics of patients with and without ADR-related admissions).

**TABLE 1:** Baseline characteristics of participants (survived at the discharge index) included in LOS and readmission analyses.

Characteristics	Unmatched			Matched		
	Total population (n = 13,569)			Total population (n = 4,674)		
	With one or more ADR-related hospital admission	Without ADR-related hospital admission	SMD	With one or more ADR-related hospital admission	Without ADR-related hospital admission	SMD
	(2,555 (18.8%))	(11,014(81.2%))		(2,337 (50%))	(2,337(50%))	
	n (%)	n (%)		n (%)	n (%)	
Age (years) (median (IQR))	76 (71, 82)	75 (70, 82)	−0.08	76 (71, 82)	77 (71, 83)	0.03
Gender						
Female	1,074 (42%)	4,887 (44%)	0.05	1,001 (43%)	1,031 (44%)	0.03
Male	1,481 (58%)	6,127 (56%)		1,336 (57%)	1,306 (56%)	
Year of first admission						
2017–2019	1,017 (40%)	5,037 (46%)	0.12	927 (40%)	963 (41%)	0.06
2020–2021	732 (29%)	2,849 (26%)		668 (29%)	605 (26%)	
2022–2023	806 (32%)	3,128 (28%)		742 (32%)	769 (33%)	
Socioeconomic status						
Disadvantage	1,553 (61%)	6,535 (59%)	0.03	1,416 (61%)	1,428 (61%)	0.01
Advantage	1,002 (39%)	4,479 (41%)		921 (39%)	909 (39%)	
Source of hospital						
Launceston general hospital	954 (37%)	3,445 (31%)	0.14	864 (37%)	905 (39%)	0.04
Northwest regional hospital	574 (22%)	2,440 (22%)		530 (23%)	517 (22%)	
Royal hobart hospital	1,027 (40%)	5,129 (47%)		943 (40%)	915 (39%)	
Charlson comorbidity index (CCI) score	2 (1, 4)	1 (0, 2)	−0.73	2 (1, 3)	2 (1, 3)	−0.02
Diagnostic related group (DRG)	​	​	0.4	​	​	0.05
Diseases and disorders of the nervous system	248 (9.7%)	1,180 (11%)	​	232 (9.9%)	247 (11%)	
Diseases and disorders of the respiratory system	332 (13%)	1,169 (11%)		291 (12%)	272 (12%)	
Diseases and disorders of the circulatory system	340 (13%)	2,228 (20%)		331 (14%)	339 (15%)	
Diseases and disorders of the digestive system	430 (17%)	1,085 (9.9%)		365 (16%)	364 (16%)	
Diseases and disorders of the hepatobiliary system and pancreas	107 (4.2%)	421 (3.8%)		90 (3.9%)	81 (3.5%)	
Diseases and disorders of the musculoskeletal system and connective tissue	195 (7.6%)	1,549 (14%)		184 (7.9%)	177 (7.6%)	
Diseases and disorders of the skin, subcutaneous tissue and breast	74 (2.9%)	506 (4.6%)		73 (3.1%)	82 (3.5%)	
Endocrine, nutritional and metabolic diseases and disorders	179 (7.0%)	432 (3.9%)		170 (7.3%)	175 (7.5%)	
Diseases and disorders of the kidney and urinary tract	157 (6.1%)	699 (6.3%)		150 (6.4%)	161 (6.9%)	
Blood, immunological and neoplastic disorders	104 (4.1%)	210 (1.9%)		95 (4.1%)	99 (4.2%)	
Infectious and parasitic diseases	105 (4.1%)	358 (3.3%)		93 (4.0%)	85 (3.6%)	
Others	284 (11%)	1,177 (11%)		263 (11%)	255 (11%)	
Admission source	​	​	0.12	​	​	0.06
Acute	2,071 (81%)	8,404 (76%)	​	1,909 (82%)	1,963 (84%)	​
Non-acute	484 (19%)	2,610 (24%)		428 (18%)	374 (16%)	

SMD: standardised mean difference.

After PSM, 5,038 patients (2,519 in each group) were included in the in-hospital mortality analysis, and 4,674 patients (2,337 patients in each group) were included in the LOS and readmission analyses. Baseline characteristics before and after PSM are presented in Supplementary Table S2 for the full cohort, and in Table 1 for those who remained alive at index discharge.

Table 2 presents adjusted ORs for in-hospital mortality, comparing the results before and after PSM. Crude ORs are reported in Supplementary Table S3. In the unmatched cohort, the in-hospital mortality rate was 7.5% among patients with an ADR-related admission compared to 4.9% among those without any ADR-related hospital admissions. The adjusted OR for in-hospital mortality was 2.39 (95% CI: 1.96–2.91); p < 0.001. Following matching, ADR-related index hospital admissions remained associated with a 31% increase in odds of in-hospital mortality (adjusted OR = 1.31; 95% CI: 1.03–1.66; p < 0.05).

**TABLE 2:** Number of events and adjusted odds ratios (aORs) with 95% confidence intervals for in-hospital mortality at index admission among all patients, before (n = 14,345) and after PSM (n = 5,038).

Outcome	Group	Before PSM (n = 14,345)		After PSM (n = 5,038)
		Crude mortality (n, %)	Adjusted OR (95% CI); p-value	Adjusted OR (95% CI); p-value
In-hospital mortality	≥1 ADR (n = 2,761)	206 (7.5)	2.39 (1.96–2.91); p < 0.001	1.31 (1.03–1.66); p < 0.05
	Without ADR (n = 11,584)	570 (4.9)	Reference	Reference

OR, odds ratio; ≥1 ADR, Patients with at least one ADR-related hospital admission; PSM, propensity score matching.

Table 3 summarises the association between ADR-related hospital admissions and LOS, as well as all-cause re-hospitalisation at 30-, 60- and 90-day post-index admission discharge. LOS was significantly increased in patients with an ADR-related hospital admission (pre-matching median [IQR] 6.0 [3.0–12.0] days) compared with that of patients without any ADR-related hospital admissions (pre-matching median [IQR] 3.0 [2.0–8.0] days) (p < 0.001). Those with ADRs had a significantly longer LOS, with a ratio of 1.35 (95% CI: 1.24–1.45; p < 0.001) in the unmatched cohort and 1.24 (95% CI: 1.13–1.37; p < 0.001) in the matched cohort (Table 3).

**TABLE 3:** Median length of stay with corresponding ratios and crude mortality (n, %), hazard ratios (HRs) with 95% confidence intervals for all-cause rehospitalisation among patients who remained alive at the index discharge, before (n = 13,569) and after PSM (n = 4,674).

Outcomes	Cohort	≥1 ADR	Without ADR	Ratio (95% CI); p-value
		Median (Q1, Q3) (days)		
Length of stay	Before PSM	6 (3, 12)	3 (2, 8)	1.35 (1.24–1.45); p < 0.001
	After PSM	6 (3, 12)	4 (2, 10)	1.24 (1.13–1.37); p < 0.001
​		Crude event (n, %)		Adjusted hazard ratio (95% CI); p-value
All-cause rehospitalisation				
Within 30 days	Before PSM	532 (19.3)	1,170 (10.6)	1.29 (1.15–1.45); p < 0.001
	After PSM	474 (20.3)	296 (12.7)	1.27 (1.08–1.48); p < 0.05
Within 60 days	Before PSM	779 (28.2)	1,683 (14.5)	1.36 (1.23–1.49); p < 0.002
	After PSM	696 (25.2)	428 (18.3)	1.31 (1.15–1.49); p < 0.001
Within 90 days	Before PSM	917 (33.2)	2,086 (18.9)	1.31 (1.19–1.43); p < 0.001
	After PSM	826 (35.3)	515 (22.0)	1.29 (1.14–1.45); p < 0.001

≥ 1 ADR, Patients with at least one ADR-related hospital admission; PSM, propensity score matching; LOS, length of stay.

Readmission rates were consistently higher among patients with ADR-related hospitalisations. In the unmatched cohort, absolute rates at 30, 60, and 90 days were 19.3%, 28.2% and 33.2%, respectively. Adjusted HRs from both the unmatched and matched cohorts demonstrated consistent and statistically significant associations, with ADR-related hospital admissions linked to a 27%–31% increase in risk of rehospitalisation at 30, 60 and 90 days after index discharge (Table 3). In the post-matching analysis, the strongest association was observed at 60 days post-discharge, where ADR-related hospital admissions were associated with a 31% increase in hazard (HR = 1.31 (1.15–1.49); p < 0.001).

**FIGURE 2:**
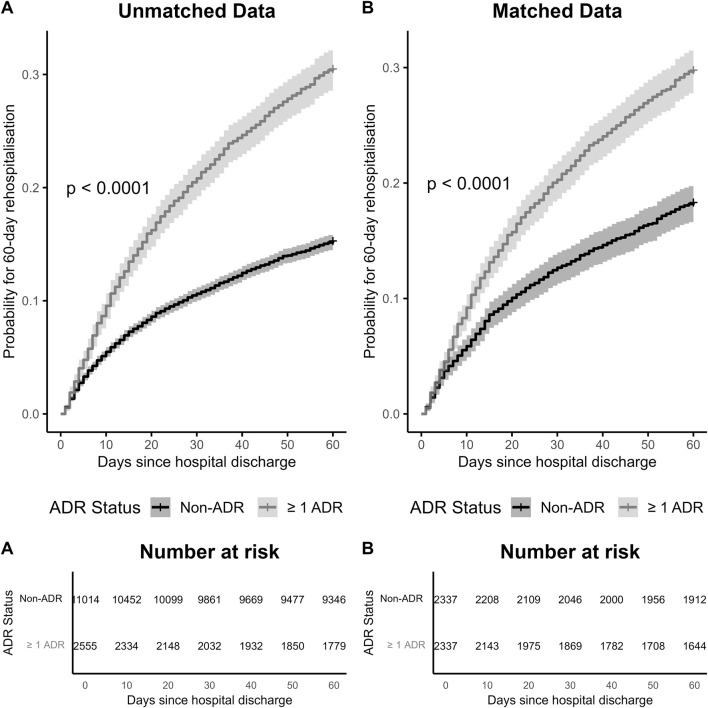
Kaplan-Meier Curves for 60-Day Readmission in older patients with diabetes, included 13,569 patients who remained alive at the index discharge. **(A)** Unmatched patients, before PSM. **(B)** Matched patients, after PSM.

Kaplan-Meier curves (Figure 2) showed that among both matched and unmatched cohorts, patients who had an ADR-related hospital admission experienced significantly higher cumulative incidence for 60-day readmission, compared to those without any ADR-related admissions. The difference was statistically significant (p < 0.0001).

## 4 Discussion

In this population-based study, nearly one in five older adults with diabetes and hospital admissions experienced at least one ADR-related admission over a period of 6.5 years. Importantly, these patients had worse outcomes than patients without any ADRs.

We applied PSM to minimise confounding between the study and control groups, resulting in well-matched cohorts across demographic, clinical, and hospital-related characteristics. We found ADR-related hospital admissions were associated with an increased length of stay and in-hospital mortality, and readmission at 30, 60 and 90 days, either before or after PSM. However, the magnitude of the associations was lower after PSM and adjustment, particularly for in-hospital mortality analysis, confirming the importance of using propensity score methods to reduce confounding bias and increase the precision of the analysis (Elze et al., 2017; Han et al., 2022).

Older adults with diabetes are inherently at greater risk of ADRs due to multimorbidity, complex medication regimens, and altered pharmacokinetics associated with ageing (Remelli et al., 2022; Elsayed et al., 2025). Our findings indicated that ADR-related hospital admissions were associated with a 2.4-fold increase in the odds of in-hospital mortality among older adults with diabetes, compared to those without ADR-related hospital admissions. This association remained significant even after adjusting for key confounders such as age, sex, comorbidities, and admissions characteristics. This finding aligns with prior research showing that diabetes itself heightens the risk of mortality following an ADR by approximately 55% (Chinmayee et al., 2024), and that individuals with diabetes have nearly triple the in-hospital mortality rate of the general population (Gao et al., 2021). The additional mortality risk observed in our cohort likely reflects the cumulative effect of diabetes-related vulnerability and medication burden. This may be explained by the complexity of diabetes management, particularly in older adults, which relates to multiple comorbid conditions and more medications. These findings reinforce the importance of medication safety and early detection of ADRs in this population. Comparable findings were also reported in a Tasmanian cohort of older adults with dementia, where ADR-related admissions similarly increased the likelihood of adverse outcomes (Zaidi et al., 2024).

Older patients with diabetes and at least one ADR had significantly longer hospital stays than those without any ADRs. This difference was observed both in unmatched and matched groups, with hospital stay being 35% longer in the unmatched group and 24% longer in the matched group. In the unmatched cohort, the difference corresponded to an approximately 3-day longer hospital stay for patients with ADRs. Similarly, a previous prospective study of acutely ill, hospitalised older patients reported significantly longer stays for those with ADRs compared to patients without ADRs (mean 12.4 ± 11.6 days vs. 7.6 ± 6.9 days; log-rank p < 0.0001) (Sandoval et al., 2021). Extended LOS is known to be associated with increased healthcare costs and resource utilisation. Beyond its impact on the healthcare system, extended hospital stay may also be associated with negative effects on patients, which includes deterioration in physical health (e.g., increased risk of opportunistic infections), impaired emotional wellbeing (e.g., prolonged separation from family), and economic burden (e.g., potential out-of-pocket expenses) (Rocha et al., 2020; Alzahrani, 2021; Hirani et al., 2025).

Using the survivor cohort, our results demonstrated that patients with ADR-related hospital admissions had consistently higher rates and hazards of readmission at 30, 60, and 90 days compared to those without ADRs. The adjusted hazard ratios before and after PSM were similar, indicating robustness of the association, with the highest risk was observed at 60 days. A previous study in older adults in France reported that about 50% of patients with ADR-related hospitalisation had all-cause readmission during the 1-year follow-up (Jang et al., 2024). Given that our study had a shorter follow-up period, the readmission rates we observed (e.g., 33.2% at 90 days) are substantially higher in comparison. These findings suggest that ADRs are associated with an ongoing health burden that predisposes patients to subsequent hospitalisations following their initial discharge. This highlights the need for targeted interventions, particularly prior to discharge, such as medication reconciliation, patients’ education regarding medications, and comprehensive discharge planning to reduce readmission risk in this vulnerable population.

The principal strength of this study lies in its large, population-based sample encompassing all major public hospitals in Tasmania and the use of a standardised administrative dataset, ensuring comprehensive coverage and high external validity. The application of propensity score matching and adjusted models helped reduce confounding bias. However, several limitations must be acknowledged. We recognised that residual confounding remained from unmeasured covariates, such as frailty index and diabetes control, which are known to significantly influence mortality and morbidity risks in older adults with diabetes (Sinclair and Abdelhafiz, 2022; Jang et al., 2024). We were unable to access these data, which likely contributes to residual confounding. Additionally, the generalisability of our findings may be limited, as our data were restricted to one state in Australia.

## 5 Conclusion

This study demonstrates that older adults with diabetes who experience an ADR face a higher risk of in-hospital death, prolonged stays in hospital, and consistently elevated readmission risk across 30, 60 and 90 days. These findings emphasise the urgent need for proactive strategies to improve medication safety in community settings and optimise post-discharge care in this high-risk population. For example, this can be achieved by involving a pharmacist to perform a home medication review using a trigger tool in conjunction with a patient interview. Deprescribing, undertaken by a pharmacist, physician, or multidisciplinary team, can further reduce medication-related risks (Gray et al., 2023; Zhou et al., 2023). In addition, empowering patients through targeted medication education and ongoing support may reduce the likelihood of ADRs and subsequent admissions. Collectively, these measures have the potential to improve outcomes, enhance quality of care, and reduce healthcare burden among older people living with diabetes.

## Data Availability

The data analyzed in this study is subject to the following licenses/restrictions: All electronic de-identified data was password-protected and stored within Microsoft OneDrive on the UTAS College of Health and Medicine Server. The Tasmanian Department of Health provided a password-protected file listing the patient names and ID numbers for elderly patient admissions between 1 July 2017 and 31 December 2023. The research team added a study number for each individual (to enable tracing). The research data was archived in the UTAS Research Data Portal (RDP) after the completion of the research project. The RDP is noted as being encrypted and is recommended by UTAS to archive the research data (https://rdp.utas.edu.au/#/). The data is accessible to only the researchers listed on the ethics application and retained for five years after publication or longer (until it no longer has research value). After this time, access to the data will be removed, making the data inaccessible and unusable. Requests to access these datasets should be directed to a.vonna@utas.edu.au.
