# Supplementary material for: Clinical outcomes of adverse drug reaction-related hospital admissions in older adults with diabetes
Source: Front Pharmacol. 2026 Jan 12;16:1729848. doi: 10.3389/fphar.2025.1729848 (PMC12832289; doi:10.3389/fphar.2025.1729848)
Supplement: Supplementary file 1 [file Supplementaryfile1.docx]

Supplementary Material

# Supplementary Table S1. The Strengthening the Reporting of Observational studies in Epidemiology (STROBE) checklist for cohort studies

|  | **Item No** | **Recommendation** |  | **Reported (Yes/No)** |  |
| --- | --- | --- | --- | --- | --- |
| **Title and abstract** | 1 | (*a*) Indicate the study’s design with a commonly used term in the title or the abstract |  | Yes |  |
|  |  | (*b*) Provide in the abstract an informative and balanced summary of what was done and what was found |  | Yes |  |
| **Introduction** | | |  |  |  |
| Background/rationale | 2 | Explain the scientific background and rationale for the investigation being reported |  | Yes |  |
| Objectives | 3 | State specific objectives, including any prespecified hypotheses |  | Yes |  |
| **Methods** | | |  |  |  |
| Study design | 4 | Present key elements of study design early in the paper |  | Yes |  |
| Setting | 5 | Describe the setting, locations, and relevant dates, including periods of recruitment, exposure, follow-up, and data collection |  | Yes |  |
| Participants | 6 | (*a*) Give the eligibility criteria, and the sources and methods of selection of participants. Describe methods of follow-up |  | Yes |  |
|  |  | (*b*) For matched studies, give matching criteria and number of exposed and unexposed |  | Yes |  |
| Variables | 7 | Clearly define all outcomes, exposures, predictors, potential confounders, and effect modifiers. Give diagnostic criteria, if applicable |  | Yes |  |
| Data sources/ measurement | 8* | For each variable of interest, give sources of data and details of methods of assessment (measurement). Describe comparability of assessment methods if there is more than one group |  | Yes |  |
| Bias | 9 | Describe any efforts to address potential sources of bias |  | Yes |  |
| Study size | 10 | Explain how the study size was arrived at |  | N/A |  |
| Quantitative variables | 11 | Explain how quantitative variables were handled in the analyses. If applicable, describe which groupings were chosen and why |  |  |  |
| Statistical methods | 12 | (*a*) Describe all statistical methods, including those used to control for confounding |  | Yes |  |
|  |  | (*b*) Describe any methods used to examine subgroups and interactions |  | N/A |  |
|  |  | (*c*) Explain how missing data were addressed |  | N/A |  |
|  |  | (*d*) If applicable, explain how loss to follow-up was addressed |  | N/A |  |
|  |  | (*e*) Describe any sensitivity analyses |  | N/A |  |
| **Results** | | |  |  |  |
| Participants | 13* | (a) Report numbers of individuals at each stage of study—eg numbers potentially eligible, examined for eligibility, confirmed eligible, included in the study, completing follow-up, and analysed |  | 4–5 |  |
|  |  | (b) Give reasons for non-participation at each stage |  | 4–5 |  |
|  |  | (c) Consider use of a flow diagram |  | 4 |  |
| Descriptive data | 14* | (a) Give characteristics of study participants (eg demographic, clinical, social) and information on exposures and potential confounders |  | 4 |  |
|  |  | (b) Indicate number of participants with missing data for each variable of interest |  | N/A |  |
|  |  | (c) Summarise follow-up time (eg, average and total amount) |  | 4–5 |  |
| Outcome data | 15* | Report numbers of outcome events or summary measures over time |  | 4–5 |  |
| Main results | 16 | (*a*) Give unadjusted estimates and, if applicable, confounder-adjusted estimates and their precision (eg, 95% confidence interval). Make clear which confounders were adjusted for and why they were included |  | 4–5 |  |
|  |  | (*b*) Report category boundaries when continuous variables were categorized |  |  |  |
|  |  | (*c*) If relevant, consider translating estimates of relative risk into absolute risk for a meaningful time period |  | N/A |  |
| Other analyses | 17 | Report other analyses done—eg analyses of subgroups and interactions, and sensitivity analyses |  | N/A |  |
| **Discussion** | | |  |  |  |
| Key results | 18 | Summarise key results with reference to study objectives |  | 5 |  |
| Limitations | 19 | Discuss limitations of the study, taking into account sources of potential bias or imprecision. Discuss both direction and magnitude of any potential bias |  | 6 |  |
| Interpretation | 20 | Give a cautious overall interpretation of results considering objectives, limitations, multiplicity of analyses, results from similar studies, and other relevant evidence |  | 5–6 |  |
| Generalisability | 21 | Discuss the generalisability (external validity) of the study results |  | 5–6 |  |
| **Other information** | | |  |  |  |
| Funding | 22 | Give the source of funding and the role of the funders for the present study and, if applicable, for the original study on which the present article is based |  | 7 |  |

*Give information separately for exposed and unexposed groups.

# Supplementary Table S2. Baseline characteristics of participants included in in-hospital mortality analysis.

|  |  | **UNMATCHED** | | | **MATCHED** | | |
| --- | --- | --- | --- | --- | --- | --- | --- |
|  |  | **Unmatched patients (n=14,345)** | | | **Matched patients (n=5,038)** | | |
| **Characteristics** | | **With one or more ADR-related hospital admission** | **Without ADR-related hospital admission** | **SMD** | **With one or more ADR-related hospital admission** | **Without ADR-related hospital admission** | **SMD** |
|  |  | **(2,761(19.2%))** | **(11,584(85.8%))** |  | **(2,519 (50%))** | **(2,519(50%))** |  |
|  |  | **n (%)** | **n (%)** |  | **n (%)** | **n (%)** |  |
| Age (years) (median (IQR)) | | 76 (71, 82) | 76 (70, 82) | -0.06 | 77 (71, 82) | 76 (71, 83) | 0.01 |
| Gender | |  |  |  |  |  |  |
|  | Female | 1,156 (42%) | 5,148 (44%) | 0.05 | 1,067 (42%) | 1,060 (42%) | 0.01 |
|  | Male | 1,605 (58%) | 6,436 (56%) |  | 1,452 (58%) | 1,459 (58%) |  |
| Year of first admission | |  |  |  |  |  |  |
|  | 2017-2019 | 1,092 (40%) | 5,286 (46%) | 0.12 | 1,011 (40%) | 1,007 (40%) | 0.02 |
|  | 2020-2021 | 794 (29%) | 2,999 (26%) |  | 707 (28%) | 727 (29%) |  |
|  | 2022-2023 | 875 (32%) | 3,299 (28%) |  | 801 (32%) | 785 (31%) |  |
| Socioeconomic status |  |  |  |  |  |  |  |
|  | disadvantage | 1,672 (61%) | 6,801 (59%) | 0.04 | 1,521 (60%) | 1,504 (60%) | 0.01 |
|  | advantage | 1,089 (39%) | 4,783 (41%) |  | 998 (40%) | 1,015 (40%) |  |
| Source of Hospital |  |  |  |  |  |  |  |
|  | Launceston General Hospital | 1,020 (37%) | 3,616 (31%) | 0.14 | 924 (37%) | 945 (38%) | 0.02 |
|  | Northwest Regional Hospital | 615 (22%) | 2,530 (22%) |  | 565 (22%) | 560 (22%) |  |
|  | Royal Hobart Hospital | 1,126 (41%) | 5,438 (47%) |  | 1,030 (41%) | 1,014 (40%) |  |
| Charlson Comorbidity Index (CCI) score | | 2 (1, 4) | 1 (0, 2) | -0.75 | 2 (1, 4) | 2 (1, 4) | -0.03 |
| Diagnostic Related Group (DRG) | |  |  | 0.4 |  |  | 0.05 |
|  | Diseases and Disorders of the Nervous System | 267 (9.7%) | 1,280 (11%) |  | 257 (10%) | 261 (10%) |  |
|  | Diseases and Disorders of the Respiratory System | 382 (14%) | 1,253 (11%) |  | 327 (13%) | 335 (13%) |  |
|  | Diseases and Disorders of the Circulatory System | 357 (13%) | 2,320 (20%) |  | 350 (14%) | 323 (13%) |  |
|  | Diseases and Disorders of the Digestive System | 464 (17%) | 1,132 (9.8%) |  | 385 (15%) | 374 (15%) |  |
|  | Diseases and Disorders of the Hepatobiliary System and Pancreas | 132 (4.8%) | 445 (3.8%) |  | 103 (4.1%) | 104 (4.1%) |  |
|  | Diseases and Disorders of the Musculoskeletal System and Connective Tissue | 204 (7.4%) | 1,598 (14%) |  | 196 (7.8%) | 200 (7.9%) |  |
|  | Diseases and Disorders of the Skin, Subcutaneous Tissue and Breast | 74 (2.7%) | 518 (4.5%) |  | 74 (2.9%) | 79 (3.1%) |  |
|  | Endocrine, Nutritional and Metabolic Diseases and Disorders | 183 (6.6%) | 449 (3.9%) |  | 174 (6.9%) | 191 (7.6%) |  |
|  | Diseases and Disorders of the Kidney and Urinary Tract | 171 (6.2%) | 725 (6.3%) |  | 161 (6.4%) | 166 (6.6%) |  |
|  | Blood, imunological and neoplastic disorders | 110 (4.0%) | 229 (2.0%) |  | 103 (4.1%) | 115 (4.6%) |  |
|  | Infectious and Parasitic Diseases | 116 (4.2%) | 411 (3.5%) |  | 108 (4.3%) | 100 (4.0%) |  |
|  | Others | 301 (11%) | 1,224 (11%) |  | 281 (11%) | 271 (11%) |  |
| Admission source | |  |  | 0.11 |  |  | 0.02 |
|  | Acute | 2,246 (81%) | 8,903 (77%) |  | 2,073 (82%) | 2,093 (83%) |  |
|  | Non-acute | 515 (19%) | 2,681 (23%) |  | 446 (18%) | 426 (17%) |  |

# Supplementary Table S3. Crude odds ratios (ORs) with 95% confidence intervals for in-hospital mortality at index admission among all patients, before (n=14,345) and after PSM (n=5,038).

| **Outcome** | **Group** | **Adjusted ORs (95% CI); p-value** | | |
| --- | --- | --- | --- | --- |
|  |  | **Before PSM (n=14,345)** | **After PSM (n=5,038)** | |
| In-hospital mortality | ≥ 1 ADR (n=2,761) | 1.56 (1.32–1.84); p<0.001 | 0.61 (0.49–0.75); p<0.001 |  |
|  | Without ADR (n=11,584) | Reference | Reference |  |

*OR = Odds ratio; ≥ 1 ADR = Patients with at least one ADR-related hospital admission; PSM = Propensity Score Matching*
